# A qualitative service evaluation of patient and caregiver experiences of CAR-T therapy: Recommendations for service development and implications for palliative care teams

**DOI:** 10.1177/02692163221138880

**Published:** 2022-11-25

**Authors:** Charlotte L Stenson, Jennifer Vidrine, Felicity Dewhurst, Wendy Osborne, Tobias Menne, Rachel Stocker

**Affiliations:** 1 Population Health Sciences Institute, Faculty of Medical Sciences, Campus for Ageing and Vitality, Newcastle University, Newcastle upon Tyne, UK; 2 Newcastle Upon Tyne Hospitals NHS Foundation Trust, Newcastle upon Tyne, UK; 3 St Oswald’s Hospice, Newcastle upon Tyne, UK; 4 School of Biomedical, Nutritional and Sport Sciences, Faculty of Medical Sciences, Dame Margaret Barbour Building, Newcastle University, Newcastle upon Tyne, UK

**Keywords:** Receptors, Chimeric Antigen, adoptive immunotherapy, palliative care, terminal care, qualitative research

## Abstract

**Background:**

Chimeric Antigen-Receptor-T-cell (CAR-T) therapy is a potentially life-saving treatment for refractory haematological malignancies. Internationally, CAR-T services are undergoing rapid development. Despite this, research on the lived experiences of patients receiving novel immunotherapies is limited. Little is known about their supportive care needs. Consequently, dedicated palliative and supportive care services may not be considered.

**Aim:**

To explore the patient and caregiver experience of CAR-T therapy and identify unmet needs to inform service development.

**Design:**

A qualitative longitudinal service evaluation. Sixteen interviews were conducted between December 2020 and March 2021 with patients (*n* = 10) and family caregivers (*n* = 4). Thematic analysis was underpinned by a constructivist approach.

**Setting/participants:**

All patients and caregivers attending one UK centre for CAR-T therapy were eligible. Semi-structured interviews were conducted at specific time points: prior to infusion, one month after infusion and follow-up post-treatment (5–18 months).

**Results:**

Identified themes described the unique challenges of CAR-T therapy. From the point of referral patients had a wide range of supportive care needs. Initially, this was attributed to prior receipt of multiple failed treatments. Subsequently, CAR-T side-effects impacted on quality-of-life and physical function. Significant psychological morbidity from prognostic uncertainty was described throughout. Patients and caregivers reported that a dedicated nurse specialist – an expert, consistent point of contact – was essential.

**Conclusion:**

Patients and caregivers would benefit from early and ongoing support from palliative care, allied-health professionals and psychology. As indications for CAR-T therapy expand, there is an urgent need for multi-centre studies incorporating patient-reported outcome data to ensure patient-centred service delivery.

**What is already known about the topic?**

- Chimeric Antigen Receptor T-cell (CAR-T) therapy has shown promising efficacy in relapsed/refractory large B-cell lymphomas, where prognosis was previously poor.
- Despite this, the majority of patients will still have disease progression following treatment, with the possibility of rapid deterioration and death.
- Survival and toxicity outcomes in CAR-T therapy are well-documented from controlled trials. However, patient-reported outcomes from clinical trials and real-world settings are limited.

**What this paper adds?**

- The trajectory of relapsed/refractory disease means patients have complex physical, functional and psychological needs at the point of referral to a CAR-T centre.
- A model of multidisciplinary supportive care, with holistic symptom management alongside parallel planning, is fundamental to ensure patients and families are supported through survivorship and end-of-life care as indicated.

**Implications for practice, theory or policy**

- The provision of multidisciplinary supportive care including a dedicated nurse specialist, psychology and palliative care are essential to address the holistic needs of patients in CAR-T services internationally.
- ‘Goals of care’ discussions are vital to comprehensively address patient expectations and support patients and caregivers in living with prognostic uncertainty.

## Background

Immune and targeted therapies are a rapidly expanding field in oncology, fostering renewed hope alongside greater prognostic uncertainty.^1^ This carries significant implications for palliative care teams, particularly with the advocation of early, integrated services. Chimeric Antigen Receptor T-cell (CAR-T) therapy is a novel immunotherapy which involves modifying patients’ own T-cells. It has shown promising efficacy in relapsed/refractory B-cell lymphomas where median survival was previously measured in months.^2^ However, durable complete response rates are only 30%–40%.^3,4^ Severe toxicity occurs in 10%–40% of patients and can be life-threatening.^5^ The treatment pathway is complex; T-cells are collected, modified and re-infused into the patient after conditioning chemotherapy. Patients have a minimum 2-week admission and must be within an hour of the treating centre for 4 weeks. Collaborative palliative care has been suggested to meet the holistic needs of patients and carers.^6^

Patient-reported outcomes (PROs) may be a better indicator of treatment toxicity than clinician-reported outcomes and are vital when evaluating treatment efficacy.^7,8^ Longitudinal patient experience data in CAR-T therapy is limited.^9^ Patients report the highest symptom burden in the first 90 days after treatment, with an association between the degree of treatment toxicity and persistence/severity of symptoms.^10^ Fatigue, poor appetite, pain and cognitive impairment are the most commonly reported symptoms, alongside reduction in physical function.^10–13^ Most patients report long-term neuropsychiatric consequences, including depression, anxiety and cognitive impairment.^11,12,14^ Perspectives from patients who fail to respond or progress rapidly following treatment are lacking.^6^

To comprehensively evaluate patient experience and inform service development, qualitative methods must be used alongside acquisition of outcome-driven data.

## Method

### Aim

To explore patient and caregiver experiences of CAR-T therapy to identify unmet needs and areas for service development.

### Study design

A qualitative, longitudinal service evaluation underpinned with a constructivist paradigm, using semi-structured interviews.^15^

### Ethical approval

This service evaluation received ethical and governance approvals from Newcastle-Upon-Tyne Hospitals Research and Development (ref: 10563). HRA approvals were not required as they deemed this to be a single-site service evaluation.

### Population and setting

**Figure 1.**
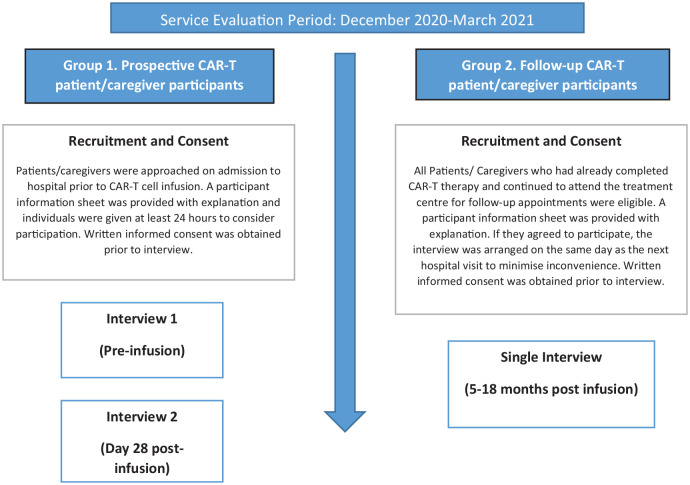
Participant groups and interview timeline.

Participants were recruited from two groups (Figure 1). All participants who fulfilled the criteria were successfully invited, recruited and consented. CS conducted the interviews in-person. They lasted 15–60 min and were audio-recorded and transcribed verbatim.

### Data collection

Inductive thematic analysis was used to evaluate the dataset.^16^ A single researcher (CS) analysed all the transcripts whilst FD and RS analysed 25% each. All researchers refined the generated themes together.

## Results

### Demographics

Sixteen semi-structured interviews were conducted with ten patients and four caregivers (Table 1). All participating caregivers were the patients’ spouse/partner. Recruitment continued until a demographically diverse patient sample and data saturation were achieved.^17^

**Table 1.** Summary of patient demographics and main themes/sub-themes.

Patient characteristics	
Age (mean (range))	54 (28–72)
Age groups	Number of patients
25–34	2
35–44	0
45–54	1
55–64	2
65–74	3
Gender	
Male (*n*)	4
Female (*n*)	6
Disease subtype	
Diffuse large B cell lymphoma (*n*)	9
Primary mediastinal B-cell lymphoma (*n*)	1
Key themes	Sub-themes
The journey to CAR-T therapy	Cycles of treatment and relapse
	Loss
Expectations of CAR-T therapy	CAR-T as a lifeline
	Lack of choice
	Perception of risk
Treatment experiences	Communication
	Isolation
	Toxicity
Dealing with uncertainty	Coping through positivity
	Future planning

## Findings

Four key themes were generated (Table 1).

### The journey to CAR-T therapy

Participants universally described CAR-T therapy in the wider context of their experience of lymphoma; characterised by cycles of treatment/admissions, and a sense of loss. The word ‘journey’ dominated the narrative. Terms such as ‘*rollercoaster*’ (Patient 10, Caregiver 2) and ‘*constant roll*’ (Patient 9) conveyed the loss of control and relentless nature of treatment. This was important in defining current treatment goals and expectations.

*If it comes back in four/five years’ time there will be another trial, then I’ll take that trial, get back in remission for two years, but it’s a vicious circle. But I do believe that once you’ve got cancer, you’ll never get rid of it, it will always come back in your lifetime and bite you in the backside. (Patient 1)*

The caregiver experience was also dominated by the cyclical nature of treatment.

*It’s a bit of a bubble isn’t it when you have a chronic illness, and it’s a revolving circle of going from one treatment to the next and [she] is in that bubble and I feel like I’m – I’m not on the outside – but you know, you feel sometimes out of control. (Caregiver 2)*

Participants described disruption to their daily lives and future plans, alongside a loss of normality.

*A longing for an end to all of the treatments, all of the hospital appointments, just you know to be able to go to work, have a normal life. I don’t want anything special, just some normality. (Patient 10)*

This loss extended beyond routines of daily living, to a loss of identity.

*I was always very independent. I used to have my own business and that you know, and I feel now that I’m not the person I used to be. I feel like I’ve been robbed really, but that’s not through the CAR-T, it’s through the cancer. (Patient 2)*

### Expectations of CAR-T therapy

Most prominent was the idea that CAR-T was ‘*a lifeline*’ (Patient 10). One patient described CAR-T as ‘*a euphoria in having a direction to go in, a positive direction*’ (Patient 3). Many participants also talked about hope, ‘*it lifted our hopes big time*’ (Caregiver 2). It was described as ‘*revolutionary*’ (Patient 10), and ‘*special*’ (Patient 4). This sense of a unique opportunity was important in decision-making; treatment decisions were viewed in the context of no alternative, and patients generally rejected the idea of choice.

*I don’t think I had a choice really. . .it was that or you won’t be here much longer. (Patient 6)*

The idea that CAR-T provided hope was universal, but expectations of treatment outcome varied. Some participants talked about cure, others about remission. Those who did not expect CAR-T to be curative were more likely to reflect on the possibility of treatment failure.

*To get me a bit longer. Just to get me a bit longer because without it it’s not a very good prognosis, it will be months and I’m not ready for that yet. (Patient 4)*

Patients also described the additional burden of having to mediate family or caregiver expectations. Use of statistics was helpful to inform expectations.

*When [the doctor] gave me the figure of 35-40% that made me think, well we have to be realistic here, because otherwise I’m feeling more for my carer – at least now [she] is in a mindset that it could go wrong. (Patient 8)*

Expectations of treatment toxicity were focussed on neurotoxicity and intensive care admission. For most, discussions of treatment-associated risk were viewed through a lens of having nothing to lose so this did not significantly impact decision-making.

*If she didn’t have it, she was going to die anyway so our view is even with the smallest percentage it was worth the risk to take. (Caregiver 2)*

### Treatment experiences

Experiences of CAR-T therapy were centred on communication with healthcare professionals, treatment toxicities and the inpatient experience. Responses were framed by the COVID-19 pandemic which increased perceived isolation and vulnerability.

The CAR-T nurse specialist was consistently identified as a single point of contact, vital for care continuity and treatment/service navigation.

*[The nurse specialist] phones up even if I haven’t got an appointment, asks how I am, am I putting the weight on, and that makes you feel better, you know, just in yourself. (Patient 7)*

Most patients described CAR-T therapy as a unique and challenging experience. The prolonged hospital admission and intensive monitoring were associated with a feeling of confinement.

*I felt like a caged animal. (Patient 7)*

There was significant variation in treatment tolerance; some patients had few side-effects, where others had severe toxicity requiring intensive care admission. Common side-effects were fever, fatigue, poor appetite and impaired memory/cognition.

*It turned out [the side effects] have been relatively mild, I’ve not really suffered any sort of effects on my brain functioning (Patient 5)*

Patients felt prepared for these in the inpatient phase (first 14 days) but were less prepared for prolonged side-effects after discharge. Assessment and management of symptoms continued to be necessary post-discharge, but patients had limited access to multidisciplinary support.

*I was just quite surprised at how tired she actually was. But apart from that, I expected her to be tired and I knew she’d be in pain cos she’d had pain all year, that hasn’t changed really. (Caregiver 3)*

Distance from the treating centre impacted on experience. Patients who lived more than an hour away were discharged to a hotel. This was an anxious time as patients/carers had to monitor for treatment toxicity.

*I was quite worried, being in the hotel with him, knowing things that could have happened. (Caregiver 4)*

Geographical distance continued to impact experience post-discharge.

*A lot of her time has been involved in coming down for blood tests all the time. . …it feels like she’s having less and less time at home, for me that’s how I see it. (Caregiver 2)*

### Dealing with uncertainty

The uncertainty associated with CAR-T therapy affected patients’ coping strategies and ability to plan for the future, contributing to psychological distress. Coping was strongly rooted in ‘keeping positive’. Many patients acknowledged the possibility of death or treatment failure but used positivity to build resilience.

*But it’s always at the back of your mind are you going to die? But I suppose you just have to switch off, and just keep positive. That’s what I try to do anyway, and I’ve got this far. (Patient 1)*

One patient described the pressure to maintain a positive approach.

*Everyone keeps saying to me – gosh you’re so strong you’re the strongest person I know – and I’m thinking I’m not, I just put this demeanour on that they think I am. (Patient 2)*

The sense that life was on-hold whilst awaiting the outcome of CAR-T was common. Participants reported anxieties about employment, finances and future treatments. Even with treatment response, patients described continuing to live with uncertainty.

*It’s just the thought that if I get [into remission] will it be long lasting, will I have to go down another route and can I emotionally cope with that anymore? (Patient 10)*

## Discussion

Through qualitative exploration of experiences, this service evaluation highlights the physical, functional and psychological needs of patients and their family caregivers undergoing CAR-T therapy.

Participants from this evaluation and other studies^10–12,14^ report that physical and psychological effects of CAR-T therapy are severe and often prolonged. Fatigue, poor appetite, pain and neuropsychiatric symptoms impact on quality of life and function resulting in significant changes to practical and social aspects of daily living. Psychological needs result from experiencing multiple treatments (with associated high expectations) and subsequent relapses. There is also a clear need for support with prognostic uncertainty. The possibility of acute, rapid deterioration alongside the potential for cure makes prognostication and advance care planning challenging in haematological malignancies.^18^ Key indicators of risk of deterioration and death include: age, co-morbidities, declining performance status and relapsed/refractory disease status.^19^ Patients with refractory DLBCL will usually meet several of these criteria at referral for CAR-T. Thoughtful communication around prognostication and goals of care should acknowledge uncertainty and explore the range of outcomes in parallel planning – simplified to ‘hoping for the best and preparing for the worst’.^1^ A study of bereaved carers of immunotherapy patients highlighted a lack of preparedness for deterioration and late integration of palliative care,^6^ despite evidence supporting specialist palliative care integration in similar populations.^20^ The description of CAR-T as a ‘lifeline’, and meeting uncertainty with positivity raises important questions about how palliative care might be perceived by this group.

Whilst the dedicated nurse specialist improved participant experiences by providing a single point of contact for support, co-ordination and continuity of care, the complexity of needs experienced by this group would also benefit from palliative care, psychology and allied health professionals. Input from these professionals should be routinely and proactively built into CAR-T services and the impact and acceptability of this should be assessed.

### Strengths and limitations

To our knowledge, this is among the first qualitative work exploring the experiences of CAR-T patients and caregivers at multiple points in the treatment journey, including perspectives on long-term treatment impact. Conclusions were limited by follow-up patients being biased towards survival, reflecting an issue reported in CAR-T clinical trials recording PRO data. The local nature of the service evaluation and the service-specific aspects of the findings may not be transferable.

### Recommendations

Multi-centre prospective studies incorporating PROs and qualitative interviews with long-term follow-up are needed to better understand the long-term impact of CAR-T therapy, and explore service-users’ views on the role of palliative care.

## Conclusion

CAR-T therapy is a promising development, but it brings growing care-provision challenges as many patients do not attain long-term remission. The complexity of patients’ and caregivers’ needs experienced prior to, throughout and following treatment demonstrates that CAR-T services should incorporate palliative care, psychology and allied health professionals together with a dedicated nurse specialist. There is an urgent need for multi-centre qualitative research into patient and caregiver experiences to inform patient-centred service models.
